# Population Patterns and Dynamics of *Ilisha elongata* (Clupeiformes: Pristigasteridae) Revealed by Target Enrichment Data

**DOI:** 10.1111/eva.70142

**Published:** 2025-08-06

**Authors:** Qian Wang, Jiantao Hu, Tianqin Wu, Wenhao Wang, Jie Zhang, Jin‐Koo Kim, Chenhong Li

**Affiliations:** ^1^ East China Sea Fisheries Research Institute Chinese Academy of Fishery Sciences Shanghai China; ^2^ Shanghai Universities Key Laboratory of Marine Animal Taxonomy and Evolution Shanghai Ocean University Shanghai China; ^3^ Engineering Research Center of Environmental DNA and Ecological Water Health Assessment Shanghai Ocean University Shanghai China; ^4^ Shanghai Ocean University Shanghai China; ^5^ State Key Laboratory of Animal Biodiversity Conservation and Integrated Pest Management, Institute of Zoology Chinese Academy of Sciences Beijing China; ^6^ Department of Marine Biology Pukyong National University Busan Republic of Korea

**Keywords:** fisheries management, *Ilisha elongata*, Northwestern Pacific, phylogeography, quaternary

## Abstract

The elongate ilisha ( *Ilisha elongata* ) is an important commercial species found along the Northwestern Pacific Coast. A sharp decline in the annual catch of *I. elongata* over recent decades implies a concerning situation regarding its fishery stocks. Nonetheless, inadequate knowledge of the genetic diversity, population structure, and historical demography of this species has hindered the establishment of sustainable fishery policies and appropriate conservation measures. In this study, the genetic structure and population demography of *I. elongata* stocks along the Northwestern Pacific Coast were examined using target‐gene enrichment data from 144 *I. elongata* individuals collected from 18 locations. The analysis revealed an average heterozygosity value of 0.2321 across variable sites in all *I. elongata* populations. Furthermore, inter‐population differentiation is relatively low, with most geographical populations displaying minimal genetic distinctions or none from one another. Population clustering analysis identified four lineages of *I. elongata* stocks. Through historical demography simulations, it was proposed that the Yalu River Estuary population diverged initially around 32,802 generations before present, while the remaining lineage split into two about 9120 generations ago. One lineage represents the southern population, while the other further separated into the northern population and the Japanese population approximately 4200 generations ago. Furthermore, secondary contact between the southern and northern population was evidenced by either population clustering or demography simulation results. These results underscore that the current phylogeographic patterns of *I. elongata* may result from directional selection due to low temperature and geographic barriers during and post glacial periods.

## 1 Introduction

Global changes are leading to a decline in genetic diversity, with a 6% decrease in wild organisms worldwide over the last two centuries (Leigh et al. 2019). This loss of biodiversity weakens a species' resilience and ability to adapt to reductions in population size and environmental changes, potentially leading to the depletion of population resources and even species extinction (Allendorf and Hard 2009). Commercial marine fishes, such as the pelagic and in‐shore fish elongate ilisha ( *Ilisha elongata* ), are among those affected, showing reduced genetic diversity or lower levels of genetic differentiation between intraspecific populations compared to non‐economically important species, as reported in previous studies (Pinsky and Palumbi 2014). The global collapse of fisheries due to overexploitation and climate change poses a crisis, emphasizing the need to preserve fish genetic diversity and protect locally adapted populations. Understanding the population genetic structure of marine fishes, including *I. elongata* , is crucial for effective long‐term management and conservation strategies of fishery stocks.

Marine fishes generally exhibit a limited genetic differentiation among populations compared to freshwater and anadromous fishes, attributed to high oceanic connectivity and large effective population sizes that reduce genetic drift (Nielsen et al. 2009). However, significant genetic diversity among populations has been observed at various scales, ranging from cross‐oceanic distances (Díaz‐Arce et al. 2024) to small geographical patterns within tens of kilometers (Carreras et al. 2017). This inter‐population genetic differentiation is driven by factors such as local selection and adaptation to specific environments (Carreras et al. 2017) or biological traits like regional philopatry (Swift et al. 2023), which can restrict gene flow.

The elongate ilisha fish, targeted for fisheries, is native to the Northwest Pacific to the Java Sea off Sarawak, inhabiting tropical, subtropical, and temperate waters along the eastern and southeastern Asian coast (Whitehead 1985; Zhang 2001). Like other clupeiform species, *I. elongata* is a migratory fish that ventures into estuaries and even freshwater for spawning during annual breeding seasons (Zhang 2001). The spawning period of *I. elongata* spans from April to mid‐September, showing variations in timing among different geographical populations (Zhang et al. 2009). Commercial and recreational fisheries targeting *I. elongata* are prevalent along the coasts of China, the Korean Peninsula, and Japan (Kim et al. 2007; Masui et al. 2016; Wang et al. 2016). Nevertheless, the total stock of *I. elongata* in the region has been steadily decreasing over the past decade (Kim et al. 2007; Wang et al. 2016). For instance, in China, the total catch of *I. elongata* in 2022 was 57,833 tons, representing only 69% of the 2012 record (CSY 2023). This decline is evident not only in catches but also in the reduced body size of harvested individuals (Chen 1988; Kim et al. 2007). Moreover, previously abundant distribution areas like the Bohai Sea have seen no official records of *I. elongata* for years (Zhang 2001). The key to reversing this concerning situation lies in establishing efficient long‐term management and conservation strategies related to local fishery stocks, which cannot be achieved without a thorough understanding of the population genetic structure of *I. elongata* along the Northwestern Pacific Coast.

Earlier studies categorized *I. elongata* stocks in the Northwestern Pacific into various geographic subgroups based on morphological traits, growth characteristics, and specific sites (Zhang and Takita 2007; Kim et al. 2007). These hypotheses lacked genetic verification and had limited geographic coverage compared to the species' broad distribution. Genetic studies on *I. elongata* population structure have been scarce and mostly confined to specific regions. Research on the mitochondrial *D‐loop* control region indicated notable genetic differentiation among Chinese coast populations, highlighting uniqueness in the Qingdao population compared to Guangzhou, Xiamen, and Zhoushan, with substantial gene flow among the latter three (Wu et al. 2009). Another study using the 16S rDNA marker reinforced these findings and emphasized the continued genetic diversity of *I. elongata* along the Chinese coast, despite challenges like overfishing (Lv et al. 2010). In contrast, a different study suggested a decline in genetic diversity in the East China Sea due to overfishing and environmental factors, based on the analysis of *COX1* and *D‐loop* loci diversity (Li, He, et al. 2013).

A previous study by Wang et al. (2016) filled gaps in the population structure and genetic diversity of *I. elongata* along the Northwestern Pacific Coast. They analyzed *D‐loop* sequences from samples collected at 16 locations across China, the Korean Peninsula, and Japan. Their findings revealed two clades: a monophyletic clade with a single population from the Yalu River estuary, and another clade indicating high gene flow among fish from the other 15 sites. Wang et al. (2016) also suggested the presence of ancestral populations in the South China Sea and proposed that the current distribution patterns of *I. elongata* could be due to population expansion or multiple colonization events. However, both their study and earlier ones by Wu et al. (2009), Lv et al. (2010) and Li, He, et al. (2013) did not offer a complete understanding of the population structure and genetic diversity of *I. elongata* along the Northwestern Pacific Coast, primarily due to limitations of mitochondrial markers. Criticism against mitochondrial markers in population genetics stems from the difference in mitochondrial DNA diversity compared to genome‐wide diversity and the maternal inheritance of mtDNA. Unlike the non‐recombining maternally inherited mitochondrial locus, nuclear genomes undergo recombination with each generation, reflecting genetic drift and enabling heterozygosity detection. The detailed demographic history and microevolutionary forces shaping the genetic diversity of *I. elongata* remain uncertain, warranting a reevaluation using genome‐wide markers to better understand its population structure and genetic diversity along the Northwestern Pacific Coast.

The study investigated the genetic structure of *I. elongata* along the Northwestern Pacific Coast using target‐gene enrichment data. By analyzing the diversity of numerous nuclear loci, the study aimed to: (1) reveal the genome‐wide genetic diversity and population structure of *I. elongata* ; (2) comprehend the demographic history of each lineage; and (3) evaluate the influence of gene flow and selection on molding the current genetic structure of *I. elongata* . The primary goal of this research is to provide valuable insights for conserving populations with unique variants that could improve the species' survival and promote a sustainable fishery.

## 2 Material and Methods

### 2.1 Sampling and Laboratory Procedures

**FIGURE 1:**
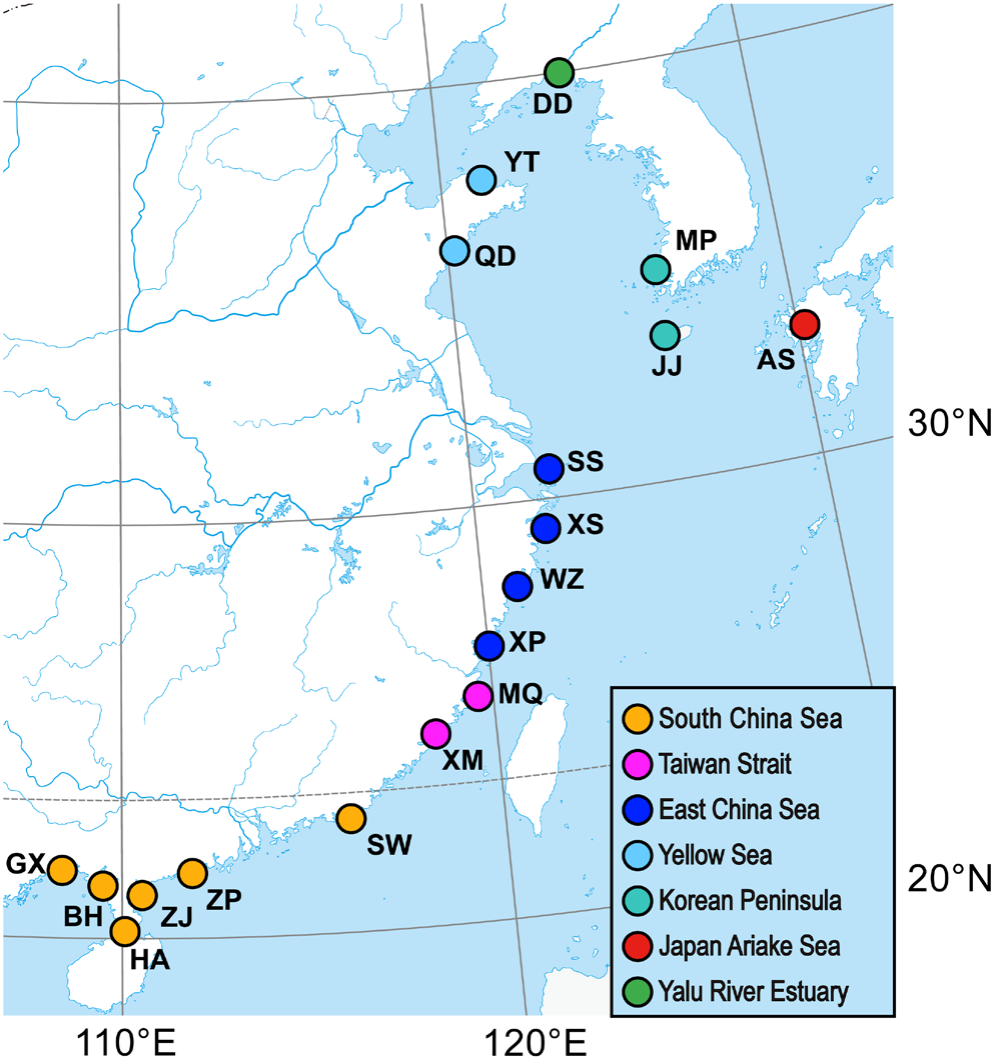
Eighteen sampling localities of 144 *Ilisha elongata* individuals across seven regions along Northwestern Pacific Coast.

Tissue samples, such as fin clips or muscle, were collected from 144 adult *I. elongata* captured at 18 coastal or estuarine localities within seven regions along the Northwestern Pacific Coast between May 2014 and October 2015 (Figure 1 and Table 1). All fishes were collected by local fishermen with trawling or line‐fishing. Tissue samples were preserved in 95% ethanol and then stored at 4°C.

**TABLE 1:** Sampling information of 144 *I. elongata* individuals.

Code	Voucher number	No. of samples	Locality	Latitude	Longitude	Regions
GX	CL1024	20	Fangchenggang, China	21°41′12″	108°21′17″	South China Sea
BH	CL1226	18	Beihai, China	21°28′52″	109°07′12″	South China Sea
HA	CL664	7	Haian, China	20°16′16″	110°13′23″	South China Sea
ZJ	CL670	1	Zhanjiang, China	21°16′23″	110°21′29″	South China Sea
ZP	CL673	3	Zhapo, China	21°34′34″	111°50′9″	South China Sea
SW	CL674	4	Shanwei, China	22°46′19″	115°21′18″	South China Sea
XM	CL678	6	Xiamen, China	24°27′43″	118°4′19″	Taiwan Strait
MQ	CL681	9	Minqing, China	26°14′13″	118°52′37″	Taiwan Strait
XP	CL1029	8	Xiapu, China	26°51′58″	119°58′44″	East China Sea
WZ	CL679	5	Wenzhou, China	28°1′23″	120°41′2″	East China Sea
XS	CL683	3	Xiangshan, China	29°28′30″	121°51′47″	East China Sea
SS	CL700	8	Shengsi, China	30°43′48″	122°26′31″	East China Sea
YT	CL1145	8	Yantai, China	37°27′49″	121°26′46″	Yellow Sea
QD	CL682	18	Qingdao, China	36°4′33″	120°21′58″	Yellow Sea
DD	CL1133, CL1134	8	Dandong, China	40°00′40″	124°21′53″	Yalu River Estuary
MP	CL963	4	Mokpo, Korea	34°48′28″	126°23′40″	Korean Peninsula
JJ	CL1031	5	Jeju‐do, Korea	32°22′10″	126°08′41″	Korean Peninsula
AS	CL1030	9	Ariake Sound, Japan	32°59′46″	130°21′1″	Japanese Archipelago

Ezup Column Animal Genomic DNA Purification Kit was used for DNA extraction following the instruction of the manufacturer (Sangon, Shanghai, China). The concentration of purified DNA was checked by a NanoDrop 3300 Fluorospectrometer (Thermo Fisher Scientific. Wilmington, DE, United States) and visualized using agarose gel electrophoresis. Fifty microliter of purified DNA was fragmented to ~250 bp using SCIENTZ18‐A Ultrasonic DNA Interrupter (Scientz, Ningbo, China). The DNA fragmentation protocol involves 22 cycles of ultrasonication for 20 s followed by a pause of 20 s, with a processing power of 300 W. One microliter of sheared DNA was load to a 1.5% agarose gel to check the size of sheared DNA fragments.

Library preparation and gene capture were carried following Meyer and Kircher (2010) with modifications (Li, Hofreiter, et al. 2013). Each sample was labeled with a pair of inline indices in the ligation step of library preparation to minimize the potential risk of cross‐contamination between samples in the subsequent gene capture steps (Wang, Yuan, et al. 2022). The DNA libraries were mixed equimolarly for the subsequent gene capture experiments. A set of biotinylated RNA‐baits (#ClupeiformsV2, Daicel Arbor Bio sciences, Ann Arbor, MI 48103, USA), designed based on 4434 single‐copy nuclear exon markers was used (Wang, Dizaj, et al. 2022). Target enrichment for all samples was preformed twice according to Li, Hofreiter, et al. (2013). Then the enriched libraries were amplified using a pair of P5 and P7 primer that both labeled with an 8 bp DNA index. The amplified products were pooled equimolarly and purified with a gel‐based fragment size selection using a QIAquick Gel Extraction Kit (Cat #28706; QIAGEN China, Shanghai, China). Purification products between 250 bp and 1000 bp were kept and then sequenced on an Illumina HiSeq 2500 sequencer by GENEWIZ (Suzhou, China).

### 2.2 Sequencing Data Filtration and Assembly

Raw reads of sequencing data were parsed according to their 8 bp indices and 6 bp inline indices. Then Trim_galore v0.6.7 (Trim_Galore 0.6.7. https://github.com/FelixKrueger/TrimGalore) and Cutadapt v1.2.1 (Martin 2011) with default parameters were used to trim the adaptor sequence and reads with low‐quality scores (*Q* < 20). Data assembly was performed according to Yuan et al. (2016). Briefly, the resulting trimmed raw reads were assembled into locus‐based output using a Perl script wrapper developed by Yuan et al. (2019). During the assembly step, putative target sequences were compared to the reference genome using BLAST, and any sequences that failed to hit the target region were considered paralogs and discarded. Additionally, loci with coverage in fewer than 70% of individuals (sample size *n* < 100) were classified as poorly enriched and excluded, while the remaining sequences were proceeded for subsequent analyses. The filtered outputs were aligned in batch using Clustal Omega (Sievers et al. 2011) based on codons.

### 2.3 SNP Calling, Population Genetic Metrics

The consensus sequences for the reference of SNP calling were generated from the aligned assembled reads using a Perl script consensu.pl. (Yuan et al. 2019) and only SNPs from coding regions were targeted. Genotyping was proceeding in a workflow as follows: The cleaned reads were mapped to consensus sequences using BWA v0.7.16a‐r1181 (Li and Durbin 2009). Subsequently, the resulting SAM files were converted into binary BAM files with Samtools v1.10 (Li et al. 2009). Picard (http://broadinstitute.github.io/picard) was used to remove PCR repeats. Finally, GATK v4.0 was employed to genotype the SNP loci and generated the resulting VCF files (Mckenna et al. 2010). SNPs were filtered with the parameters including MAF > 0.05, max‐missing set as 0.80, and min‐meANDP set as 3 using VCFtools (Danecek et al. 2011). Only one SNP from each locus was selected to avoid linkage disequilibrium. The mean pairwise population differentiation (*F*_ST_) and heterozygosity of the variable sites were calculated with 1000 bootstrap resamplings using the “populations” program in the Stacks (Catchen et al. 2013; Wright 1968). The *p*‐value to keep an *F*_ST_ measurement was set to 0.05 by default. Given that the ZJ group consists of only one individual, to ensure that the population genetic metrics results are biostatistical significant, this locality was excluded before proceeding the Stacks calculation.

### 2.4 Population Structure

Input files for population structure inference were generated from VCF files by VCFtools (Danecek et al. 2011). Population clustering was evaluated using the Bayesian clustering method‐based program STRUCTURE v2.3.4 (Pritchard et al. 2000). The STRUCTURE operating parameter was set under the model of correlated allele frequencies and a burn‐in period of 50,000 followed by 500,000 Markov Chains Monte Carlo (MCMC) reps. The analyses for estimating the number of populations were conducted with three iterations for each value of *K*, ranging from one to the number of regions plus two as suggested by Evanno et al. (2005). The most likely *K* value was determined using Structure Harvester v0.6.93 (Earl and Vonholdt 2012). The resulting three iterations with the highest ad hoc statistic Δ*K* were averaged using CLUMPP v1.1.2 (Jakobsson and Rosenberg 2007) then displayed as bar plot using GraphpadPrism v9.0 (GraphPad Software, Boston, Massachusetts USA, www.graphpad.com). PLINK1.9 (Chang et al. 2015) was applied to conduct Principal Component Analysis (PCA) for *I. elongata* on the scale of the number of regions (*n* = 7) to reveal their relationships. Three calculated PCAs were kept and visualized using R package ggplot2 v3.4.4 (Wickham 2016). As identified by Wang et al. (2016), *I. elongata* from the Yalu River estuary were genetically different from those distributed in other regions in their mitochondrial *D‐loop* locus. Hence an additional PCA with same parameters setting based on the data matrix excluding all individuals from Yalu River Estuary (DD, CL1133 and CL1134) were performed simultaneously to examine the genetic relationship of *I. elongata* distributing in the rest of six regions. In addition, PCA was conducted for locations with a sample size exceeding 5. This was employed to mitigate potential biases in population clustering arising from variations in sample size across locations and to improve the robustness and reliability of the results.

### 2.5 Demographic History Simulation

According to our population clustering result and geographical regions, fishes from 18 localities could be lumped into four clades and noted as pop 0 (GX to ZP), pop 1 (SW to YT), pop 2 (AS) and pop 3 (DD). Based on the divergence scenario of three populations proposed by Wang et al. (2016), we hypothesized three models (Model 1–3) to test the divergence sequence of these four populations (Figure S1). Model 1 and 2 hypothesizes that the divergence event between pop 2 and pop 0 occurred prior or posterior to that between pop 1 and pop 0, while model 3 proposes that pop 2 emerged as a sub‐divergent split from pop 1 following the separation between pop 1 and pop 0. All simulations under these models were conducted under the scenario of strict isolation (SI). The remaining migration models are extensions of the best‐fit model identified in the previous analysis, incorporating allowances for gene flow between geographically adjacent populations (Figure S1). As indicated by the population clustering, we considered three alternative migration scenarios between pop 0 and pop 1: Isolation‐with‐Migration (IM, model 4), Ancient Migration (AM, model 5), and Secondary Contact (SC, model 6) that were initially developed and used in Rougeux et al. (2017). Meanwhile within the above three models, pop 1 exported immigrants to neighboring pop 2 and pop 3 steadily, and vice versa. The best migration model would rerun with addition parameters that enable temporal variation in the effective population size. Considering the age at first maturity for *I. elongata* along the Northwest Pacific coast is 2–3 years (Zhang 2001), the generation time was assumed to be 2.5 years herein. The mutation rate was assumed to be 2.5e‐8 per site per generation according to Wang, Dizaj, et al. (2022).

FASTSIMCOAL 2.8 (Excoffier et al. 2021) was employed to test all hypothesized models. By using the inter‐population joint site frequency spectrum (JSFS) as a summary statistic and simulating historical events, FASTSIMCOAL2 can infer the population history such as population resizing, population divergence and inter‐population migration. The VCF file was converted into *.arp file using PGDSpider 2.1.1.5 (Lischer and Excoffier 2012). The result of the Arp file was used as the input file for ARLEQUIN 3.5 (Excoffier and Lischer 2007) to calculate the joint site frequency spectrum (JSFS). Based on the calculated JSFS file (*.obs), different evolution scenarios were simulated in FASTSIMCOAL 2.8. Independent point estimation runs for each model was 100, including 100 K coalescent simulation, and 30 ECM cycles. Each run started with the random seeds from the parameter file *.est. The optimal JSFS observed by the model with different numbers of parameter was compared with the Akaike information criterion (AIC). The conditional interval (CI) of the optimal model's point estimation was performed at 10 runs per bootstrapped JSFS. The resulting bestlhoods file contains two likehood values: the maximum possible value for the likelihood when there was a perfect fit of the expected to the observed JSFS (MaxObsLhood) and the maximum likelihood estimated base on the model parameters (MaxEstLhood). The higher the model fit with the population history, the smaller the difference between MaxObsLhood and MaxEstLhood (*L*) generated from each simulation runs (Excoffier et al. 2021).

### 2.6 Analysis of Selective Signal

The signal of selection was detected by screening the outlier sites in all population. The outlier sites were identified using two different software: ARLEQUIN 3.5 and BAYESCAN 2.1. ARLEQUIN 3.5 adopts the FDIST method (Excoffier and Lischer 2007) using coalescent simulations to create a null distribution of F‐statistics and then assign *p*‐values for each SNP based on its distributions and observed heterozygosity across all sites. The simulation was run under a hierarchical island model with the parameter of 20,000 simulations, 20 simulated groups and 100 demes per group. Furthermore, a false discovery rate (FDR) correction was applied to identify statistically significant outlier (Narum 2006). Outlier with high *F*_ST_ values would be considered to be potentially under positive selection and those with *F*_ST_ values close to zero would be regarded as candidates for balancing selection. BAYESCAN 2.1 (Foll and Gaggiotti 2008; Foll et al. 2010; Fischer et al. 2011) is designed for detecting locus under selection by implementing a Bayesian approach to generate a null distribution of *F*_ST_ for neutral loci and estimate population specific *F*_ST_ coefficients in contrast to a locus‐specific *F*_ST_ coefficient shared by all the populations. The software was executed with prior odds of 10,000 and a burn‐in of 50,000 iterations; 20 pilot runs of 5000 iterations were used to tune MCMC parameters and following 5000 sampling iterations with a thinning interval of 10, significance was evaluated using the *q*‐value, a FDR analogue of the *p*‐value, of 0.05. Loci with a positive value of alpha suggests underwent diversifying selection, whereas negative values suggest balancing or purifying selection.

Loci with outlier SNPs identified under selection with either ARLEQUIN or BAYESCAN were blasted against the genome of the Atlantic herring ( *Clupea harengus* ) (Pettersson et al. 2019), the species with a reference genome and the closest phylogenetic distance to *I. elongata* (Wang et al. 2016), using the BLASTN search tool of the Ensembl website (www.Ensemble.org). The annotated function of the gene that yielded a match with the outlier loci was searched at the UniProt database (www.uniprot.org). Finally, outliers may magnify the difference between populations. Therefore, we rerun the PCA with the outlier sites excluded, to test whether the results of population clustering and demographic history analyses were dominated by outlier loci.

## 3 Result

### 3.1 Population Genetic Diversity

In total, 10,254 SNPs from 1602 loci were identified, averaging 6.4 SNPs per locus. Following filtration, 1392 loci were kept and one SNP was randomly selected for each locus to prevent linkage disequilibrium of the data. The average heterozygosity at variant positions was 0.2321 across all localities except for ZJ, with the highest at 0.2608 for YT and the lowest at 0.1644 for DD (see Table 2). Additionally, four private alleles were detected in the DD group. Inter‐population differentiation (*F*_ST_) showed a relatively low level (Table 3), with no pronounced genetic differences (*F*_ST_ > 0.25) observed (Wright 1968). Among population pairs from 17 localities, moderate (0.15 < *F*_ST_ < 0.25) and minor (0.05 < *F*_ST_ < 0.15) genetic differentiation accounted for 7.84% and 50.33%, respectively. DD exhibited the highest divergence from other groups, with a mean *F*_ST_ of 0.1449. The greatest genetic differentiation was between DD and XS (*F*_ST_ = 0.1893), whereas the lowest was between GX and BH (*F*_ST_ = 0.0176).

**TABLE 2:** The heterozygosity of variant positions of *I. elongata* from each locality and region.

Localities	SCS mean	GX	BH	HA	ZJ^a^	ZP	SW
Value	0.2347	0.2384	0.2418	0.2362	NA	0.2194	0.2379
Localities	TWS mean	XM	MQ				
Value	0.2431	0.2511	0.2352				
Localities	ECS mean	XP	WZ	XS	SS		
Value	0.2398	0.2402	0.2466	0.2255	0.2467		
Localities	YS mean	YT	QD				
Value	0.2499	0.2608	0.2390				
Localities	KRP mean	JJ	MP				
Value	0.2214	0.2264	0.2164				
Localities	DD						
Value	0.1644						
Localities	AS						
Value	0.2194						

^a^ The ZJ group was excluded for consisting of only one individual.

**TABLE 3:** Pairwise differences (*F*_ST_, *p* < 0.05) among 17 localities; the ZJ group was excluded.

	XP	AS	JJ	DD	YT	BH	HA	ZP	SW	XM	WZ	MQ	QD	XS	SS	MP
GX	0.0322	0.0570	0.0489	0.0823	0.0356	0.0176	0.0281	0.0324	0.0422	0.0351	0.0405	0.0339	0.0340	0.0428	0.0348	0.0453
XP		0.0601	0.0516	0.1253	0.0336	0.0302	0.0483	0.0709	0.0495	0.0402	0.0482	0.0342	0.0244	0.0600	0.0375	0.0538
AS			0.0768	0.1637	0.0590	0.0566	0.0751	0.1033	0.0781	0.0686	0.0749	0.0600	0.0434	0.0856	0.0621	0.0788
JJ				0.1689	0.0497	0.0468	0.0755	0.1275	0.0734	0.0667	0.0743	0.0516	0.0317	0.1030	0.0532	0.0851
DD					0.1303	0.0823	0.1231	0.1693	0.1671	0.1443	0.1555	0.1261	0.1035	0.1893	0.1296	0.1811
YT						0.0338	0.0457	0.0658	0.0438	0.0407	0.0446	0.0341	0.0237	0.0567	0.0328	0.0510
BH							0.0256	0.0364	0.0422	0.0323	0.0406	0.0345	0.0334	0.0423	0.0343	0.0436
HA								0.0761	0.0691	0.0543	0.0675	0.0488	0.0367	0.0846	0.0492	0.0785
ZP									0.1225	0.0869	0.1061	0.0676	0.0447	0.1575	0.0707	0.1418
SW										0.0624	0.0719	0.0477	0.0277	0.1010	0.0482	0.0841
XM											0.0600	0.0428	0.0300	0.0792	0.0434	0.0679
WZ												0.0449	0.0294	0.0926	0.0489	0.0837
MQ													0.0248	0.0604	0.0346	0.0501
QD														0.0331	0.0258	0.0305
XS															0.0617	0.1122
SS																0.0517

### 3.2 Population Clustering

**FIGURE 2:**
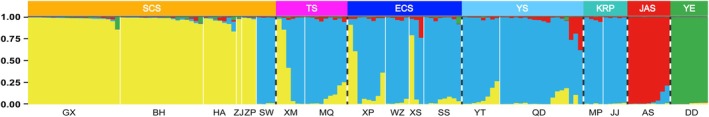
Population clustering by STRUCTURE of individual assignment to the most probable number of clusters (*K* = 4). Each individual is represented by a single bar. The different color on a single bar indicates admixture origin of the individual. Yellow denotes the southern population (SOU), blue denotes the northern population (NOR), red denotes the Japanese population (JAS) and green denotes the Yalu River Estuary population (YE). ECS, East China Sea; JA, Japanese Archipelago; KP, Korean Peninsula; SCS, South China Sea; TS, Taiwan Strait; YE, Yalu River Estuary; YS, Yellow Sea.

**FIGURE 3:**
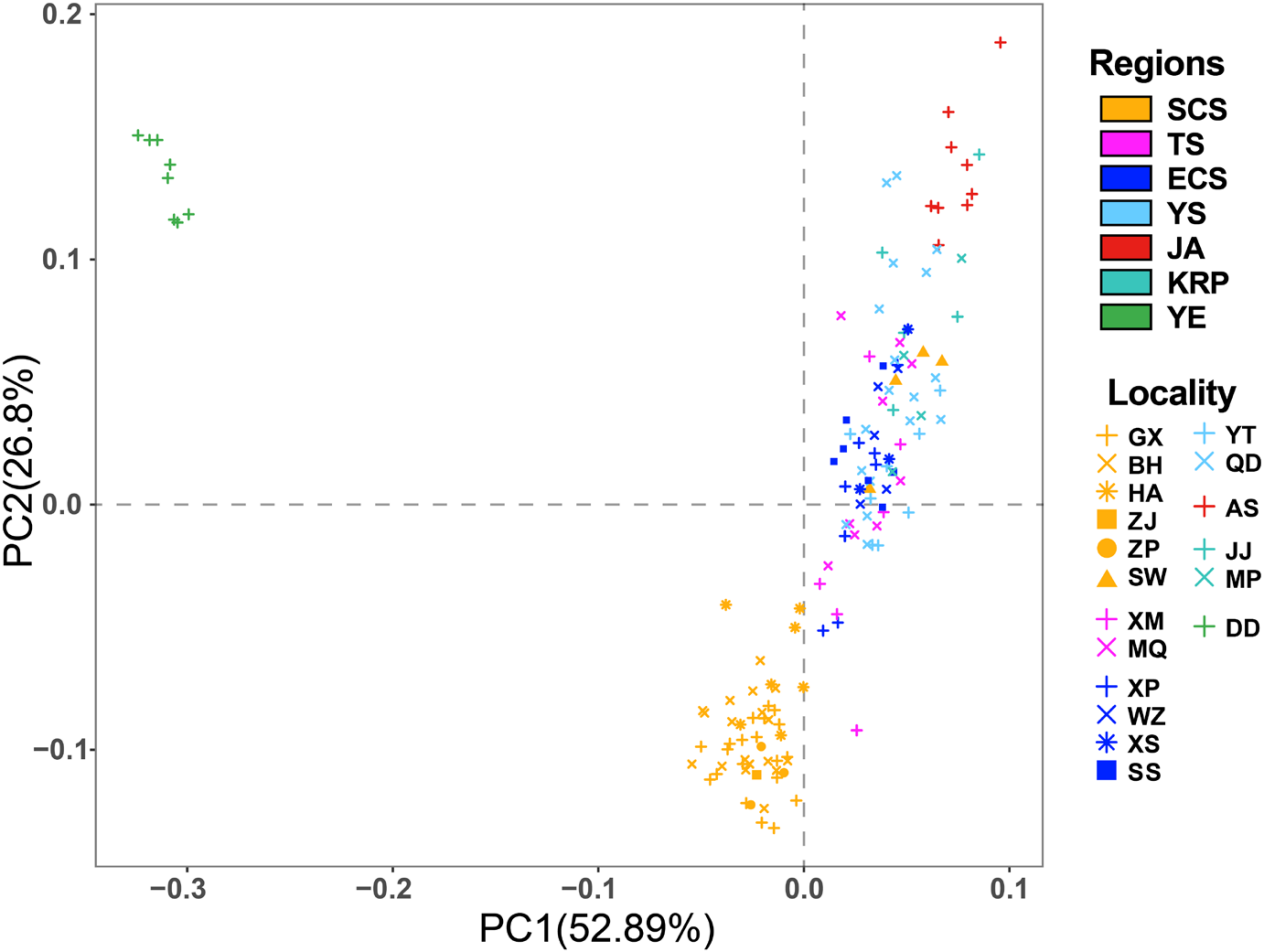
Scatterplot generated by the Principal Component Analysis (PCA). Each standalone dot denotes an individual. The color of the dot represents the geographic region, and the shape of the dot represents the sampling locality.

STRUCTURE analyses indicated that the most likely number of populations (*K*) with the highest Δ*K* value was four. The assignment probabilities for each individual (*n* = 144) to its respective populations are shown in Figure 2. All 49 fishes from five localities (GX, BH, HA, ZJ, and ZP) along the South China Sea coast, along with individuals from the Taiwan Strait (XM) and the East China Sea (XP and XS), were assigned to the southern population. The northern population comprised 73 fishes from the northern part of the South China Sea coast (SW), the Taiwan Strait (XM and MQ), the East China Sea (XP, WZ, XS, SS), the Yellow Sea (QD and YT), and the Korean Peninsula (MP and JJ). The AS group, consisting of nine individuals from the Ariake sound in the Japanese Archipelago, defined a separate cluster (Japanese population). Eight individuals collected from the Yalu River Estuary (DD) showed overall differentiation from all other localities, forming the Yalu River Estuary population. Intra‐population admixture was observed, a subset of individuals from multiple localities (XM to QD) showing predominant northern genetic proportions while also exhibiting detectable either southern or Japanese ancestry. Conversely, certain specimens within the Japanese population displayed measurable northern genetic components. Furthermore, individuals from the southern and northern populations coexisted within the groups of three localities (XM, XP, and XS) along the Taiwan Strait and East China Sea. PCA results revealed a similar genetic population structure, with fishes from the Yalu River Estuary forming a distinct cluster, while another cluster containing fishes from the remaining six regions could be subdivided into three groups (Figure 3 and Figure S2). The analysis excluding sites with fewer than 5 individuals revealed similar patterns to those observed when analyzing the complete dataset (Figure S3).

### 3.3 Demographic History

**FIGURE 4:**
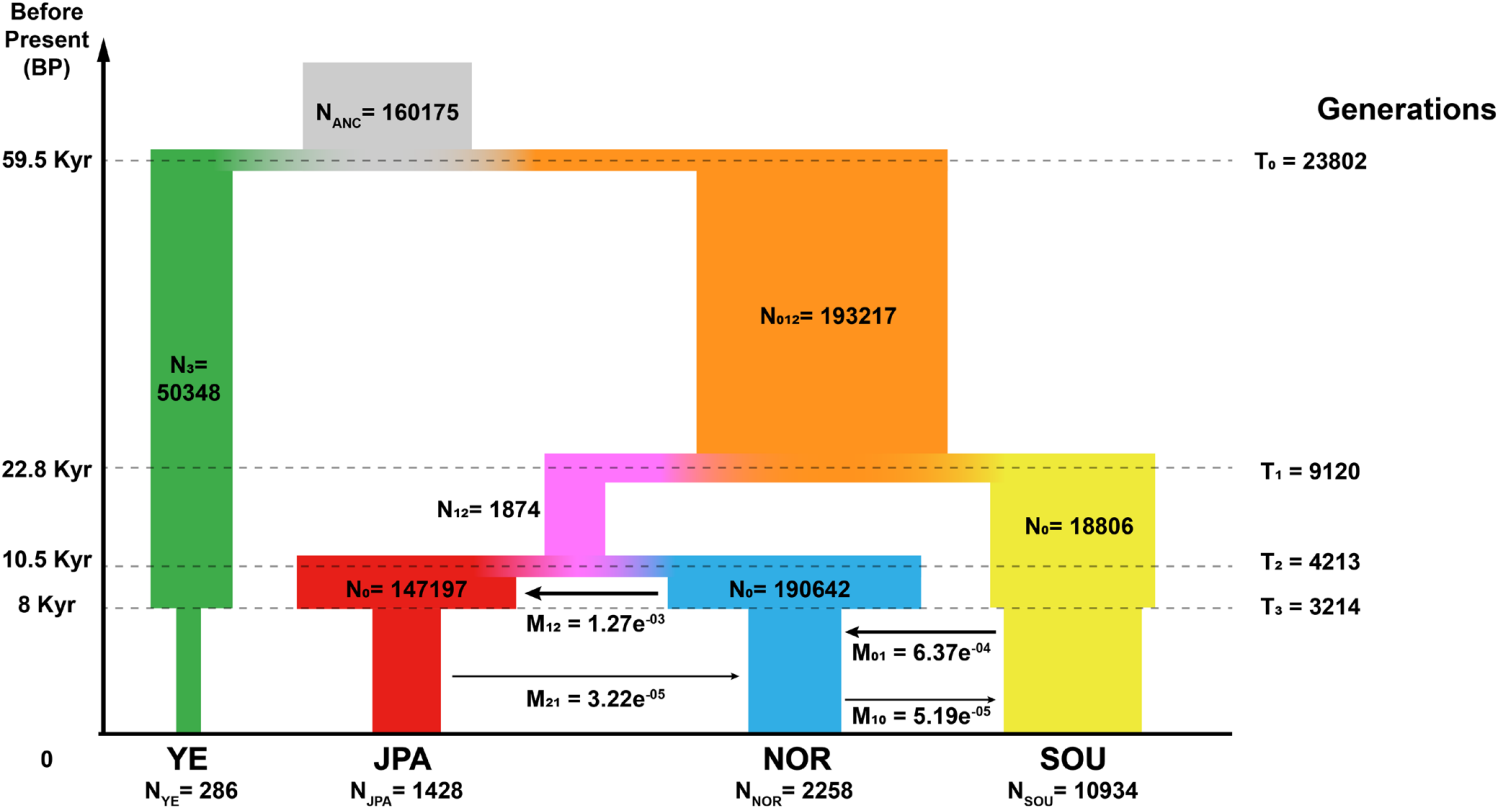
Results of simulate historical population models and estimated gene flow between adjacent populations using FASTSIMCOAL 2.8. Gray denotes the common ancestral lineage of all *I. elongata* populations, orange denotes the common ancestral lineage of southern, northern and Japanese populations, violet denotes the common ancestral lineage of northern and Japanese populations, yellow denotes the southern population (SOU), blue denotes the northern population (NOR), red denotes the Japanese population (JAS) and green denotes the Yalu River Estuary population (YE).

Four clades in demographic history simulation correspond to population clusters: the southern population (pop 0), northern population (pop 1), Japanese population (pop 2), and Yalu River Estuary population (pop 3) (Figure 4). The MaxObsLhood values obtained from FASTSIMCOAL2 analyses for all models were −5987.66. Notably, model 2 exhibited the highest *L* value (2044), whereas model 3 displayed the lowest (2018) (Table S1) with an AIC value of 36902.36. Consequently, the most likely order of differentiation for the four *I. elongata* lineages suggests the Yalu lineage diverged first, followed by the split into two lineages, with one becoming the southern lineage and the other further dividing into the northern and Japanese lineages. Therefore, migration models (models 4–6) were designed on the basis of model 3 to test different divergence scenarios. As a result, model 6 (SC model) exhibits the lowest *L* (1769) among all candidates and a comparatively lower AIC value (35778.93) than model 3 (Table S1). Therefore, this model is the most effective one in explaining the historical demography of *I. elongata* .

In accordance with model 6 (Figure 4), a portion of individuals (*N*_3_ = 50,348) split from the common ancestral population of all *I. elongata* (*N*_A_ = 160,175) approximately 23,802 generations ago (T_0_, 59.5 Kyr BP) and became the Yalu lineage. After the first divergence event, the remaining population underwent a small‐scale population expansion (*N*_012_ = 193,217) and subsequently differentiated into two lineages around 9120 generations ago (T_1_, 22.8 Kyr BP): one leading to pop 0 (*N*_0_ = 18,806) and another representing the common ancestor of pop 1 and pop 2 (*N*_12_ = 1874). It is noteworthy that both *N*_0_ and *N*_12_ are distinctly smaller than *N*_012_, indicating that this divergence event was accompanied by population contraction. Separation between pop 1 and pop 2 was estimated to have occurred 4213 generations before the present (*T*_2_, 10.5 Kyr BP). Both populations had effective population sizes (*N*_1_ = 190,642 and *N*_2_ = 147,197) markedly higher than *N*_12_, suggesting that both of them experienced demographic expansion. After the latest split event, pop 1 exported immigrants to neighboring pop 2 steadily with notable proportions (*M*_12_ = 1.27e^−03^), while gene flows in the opposite direction were weaker (*M*_21_ = 3.22e^−05^). The secondary contact between pop 0 and pop 1 dated to 3214 generations ago (*T*_3_, 8 Kyr BP); the asymmetrical gene flow suggests there was mainly northward migration (*M*_01_ = 6.37e^−04^, *M*_10_ = 5.19e^−05^). In addition, all lineages have suffered varying degrees of population contraction compared to the effective population sizes at their initial differentiation (Table S1).

### 3.4 Loci Under Selection

Sixty‐eight outliers were identified out of 1392 loci after FDR correction using the FDIST method implemented in ARLEQUIN (*p* < 0.05) across all populations (Table S2), of which 54 possessed positive *F*_ST_ values, hence considered under positive selection. The remaining 14 sites had negative values close to zero and were regarded as under balancing selection. Twenty‐five outliers (*q*‐value < 0.05) among 1392 SNPs were uncovered with BAYESCAN analysis, all of them potentially under positive selection and flagged by ARLEQUIN (Table S3).

In total, 47 sequences with SNPs identified as outliers with either ARLEQUIN or BAYESCAN were blasted against the genome of the Atlantic herring ( *Clupea harengus* ), and 39 of them match up within known genes. The functions of these genes were related to RNA metabolism, DNA damage repair, ubiquitination modification, adaptation to hypoxic environments, regulation of circadian rhythm, and tissue development (Pettersson et al. 2019). PCA results with outlier exclusion similarly support the previously identified four genetic clusters (Figure S4).

## 4 Discussion

### 4.1 Genetic Population Structure of *I. elongata*

Species characterized by high fecundity, short lifespan, wide geographical distribution, and long‐distance dispersal tend to harbor elevated genetic diversity levels (de Kort et al. 2021). As a typical *r*‐strategist, *I. elongata* produces over 60 thousand offspring per mature female individual during each spawning event and reaches sexual maturity within 2–3 years, aligning with most clupeiform species (Zhang 2001). In this study, heterozygosity levels were comparable among 16 of the 17 surveyed *I. elongata* groups, with the DD group emerging as the sole outlier showing the lowest value (Table 2). This indicates that the DD population maintains reduced genetic diversity relative to conspecific populations in other geographic regions. As genetic diversity is crucial for species' adaptive capacity and long‐term persistence, the DD group's diminished diversity suggests a more compromised population genetic structure, potentially increasing its vulnerability to environmental fluctuations and overharvesting. The overall average heterozygosity value (0.2321) was slightly below the Wright‐Fisher equilibrium prediction (Koroliouk and Koroliuk 2019), potentially associated with the excess of rare variants, indicating possible population degrowth. This observation aligns with the demographic simulation result (Figure 4).

Consistent with the prevailing notion that marine fish populations typically display limited genetic differentiation among populations, the genetic divergence among all *I. elongata* sampling groups across seven geographic regions appeared relatively low (Table 3). Most surveyed populations exhibited low pairwise genetic differentiation (0.05 < *F*_ST_ < 0.15) or little genetic distinctions (*F*_ST_ < 0.05) from others. Based on the large local census population sizes suggested by the demographic simulation, the observed *F*_ST_ levels may reflect shared selective pressures among all *I. elongata* populations. This interpretation is supported by selective signal detection results, which identified the same outlier loci across all populations. Meanwhile, four private alleles found exclusively in the DD group might account for its increased genetic differentiation from other sampling groups.

Despite minimal inter‐population differentiation among various geographic groups, our analyses unveiled that the *I. elongata* stocks along the Northwestern Pacific Coast could be classified into four genetic populations, offering enhanced resolution compared to previous mtDNA data analyses (Wang et al. 2016; Wu et al. 2009). Wang et al. (2016) proposed three lineages for *I. elongata* along the Northwestern Pacific Coast: a monophyletic lineage exclusively comprising fishes from the Yalu estuary, a basal lineage mixing individuals from many regions except for the Yalu estuary, and a more derived “north clade” lineage. Consistent with Wang et al. (2016), our population clustering analyses (Figures 2 and 3) confirmed a genetically distinct *I. elongata* lineage in the Yalu River estuary. The southern population predominantly resides in the tropical shallows of the South China Sea, while the northern population inhabits coastal waters from the south of the Taiwan Strait to the Korean Peninsula. Both populations include individuals from the “basal” lineage and “north clade” lineage as proposed by Wang et al. (2016). Surprisingly, our demographic simulation indicated that all fishes from Ariake Sound (Japan) in the clade diverged from the northern population lineage, yet both PCA and STRUCTURE analyses unmistakably supported the segregation of the Japanese population from the northern population (Figure 2 and Figure S2). Consistent with previous studies on fish native to the Northwestern Pacific Coast, the geographic distribution pattern of the four genetic populations of *I. elongata* stocks displayed a general south–north differentiation. However, recent studies on other species (*Lateolabrax maculatus* and *Setipinna tenuifilis* ) that sampled the Yalu River estuary alongside other sites across the coast of the Yellow Sea, Bohai Sea, and East China Sea did not recognize genetic distinctiveness in individuals from the Yalu River estuary (Chen et al. 2023; Liu et al. 2023). The genetic uniqueness of the Yalu River Estuary population compared to the other three populations within *I. elongata* stocks highlights the complex evolutionary trajectories this population may have undergone.

The admixture plot clearly shows the coexistence of two genetic clusters within the same locality along the coast of the Taiwan Strait and East China Sea, some with a high proportion of the southern parent and others with a high ancestry proportion of the northern parent, indicating a potential secondary contact event between two populations (Figure 2). Unsurprisingly, such divergence scenario was well supported by the demographic simulation result. This result also clearly indicated that there was a higher migration rate from the south to north against the opposite direction, which was consistent with the pattern shown in the population clustering results. It was also suggested by (Hu et al. 2022) that the recent northward dispersal of individuals of *I. elongata* from the southern population might be a factor in shaping such admixture scenario between two populations. However, prezygotic reproductive isolation between two populations due to asynchronous spawning peaks may play a key role in maintaining genetic differences between the two populations. Previous long‐term observations indicate that the spawning peaks for *I. elongata* in different regions vary. In the southern regions like the Pearl River Estuary, the predominant southern population begins spawning in March, and peaks in May (Wang et al. 2004). In contrast, in the more northerly Taiwan Strait or East China Sea, which is dominated by northern populations, the spawning start time is relatively later than that of the southern populations, with most groups reaching their peak spawning in June or July (Zhang et al. 2009).

Selection may introduce biases in population genetic diversity, differentiation, and demographic history, particularly for analyses based on coding sequences (Brandt et al. 2018; Saccheri and Hanski 2006). However, our PCA using selection‐filtered datasets resulted in a population clustering pattern (Figure S4) consistent with the original one (Figure 3). This robustness can be attributed to either all loci under selection being detected across all *I. elongata* populations or their non‐dominant proportion in the overall dataset (47 out of 1392). Nevertheless, additional analyses of non‐coding sequences should be prioritized in future work to fully characterize *I. elongata* population dynamics, thereby validating and extending our current results.

### 4.2 Phylogeography of *I. elongata*

The distribution range of *I. elongata* , spanning from the tropical coast to the temperate shallows along the Northwestern Pacific Coast, covers diverse environmental conditions. However, few studies have focused on the factors driving population differentiation or population demography. Wang et al. (2016) proposed that the South China Sea could house the ancestral lineage of current *I. elongata* stocks. According to the best model from the FASTSIMCOAL simulation (Figure 4), the ancestral lineage of *I. elongata* initially split into two demographically distinct lineages, with one lineage forming the Yalu River Estuary population and the other contributing to the remaining three populations. Given the warm‐water preference of *I. elongata* (Zhang 2001), our results support the hypothesis that the ancestral lineage of *I. elongata* likely originated from the warm waters of the South China Sea region. Like other warm‐water marine species, the common ancestral lineage of *I. elongata* in ancient South China Sea might have dispersed northward via the Kuroshio Current during the Pliocene to the middle‐late Pleistocene (Gallagher et al. 2015). Subsequently, during the Wurm Glacial stage of late Pleistocene, genetic divergence began to emerge due to changes in the flow pattern of the Kuroshio Current combined with dramatic environmental shifts in marine habitats (Xie et al. 1996; Xu 2009; Gallagher et al. 2015).

Glacial events, serving as barriers or imposing selective pressures, have been key in shaping phylogeographic patterns among marine fishes (Mach et al. 2011). As indicated by our demographical history simulation (Figure 4), the *I. elongata* stocks along the Northwestern Pacific Coast experienced multiple population differentiation events. The first event occurred approximately 59.5 Kyr BP during the Early Wurm Glaciation (MIS 4), coinciding with the peak of the global regression (Wang and Wang 1982). During this period, sea levels in the East China Sea were approximately 80 m lower than present‐day, and the South China Sea experienced similar declines in sea level, causing alterations to coastlines as well as areas of coastal shallows (Wang and Wang 1982; Liu et al. 2015). Upheavals in the marine environment led to the establishment of gene flow barriers within the common ancestral lineage of *I. elongata* , eventually resulting in the differentiation between the Yalu River Estuary population and the remaining southern population. Meanwhile, this marine regression was accompanied by a global‐scale drop in sea surface temperatures (SST) (Wang and Wang 1982). Shifts in environmental factors likely intensified positive selection within the ancestral lineage of *I. elongata* , prompting subpopulations across different geographic regions to diverge into two genetically distinct groups. Environmental factors associated with selection pressure are also evidenced by outlier sites identified within sequences that relate to adaptation to hypoxic environments and regulation of circadian rhythm.

Similarly, divergence between the southern population and the ancestral lineage of northern and Japanese populations was also shaped by glacial events. Rising sea levels and rebounding marine temperatures enabled the northward re‐expansion of all *I. elongata* populations during the Wurm Sub‐interglacial Stage (MIS 3). With the advent of the Late Wurm Glaciation (MIS 2), another large‐scale regression occurred along the Northwestern Pacific coast. By the Last Glacial Maximum (LGM, 29–18 Kyr BP), sea level in the East China Sea reached its lowest stand (−140 m below present), exposing extensive continental shelves as emergent landmasses (Wang and Wang 1982; Xie et al. 1996), which led to geographic isolation between *I. elongata* populations in different marginal seas. Simultaneously, synchronous cooling in the SST (Wang and Wang 1982; Liu et al. 2015; Xu 2009) triggered positive selection pressures on geographically separated populations under similar environmental stresses to previous glacial episodes, resulting in a homogeneity in the genetic islands between four populations as proposed by the outlier detection (Tables S2 and S3).

Unlike the previous two divergence events caused by glacial regressions, the Japanese and northern populations underwent a much more recent post‐glacial separation process. This differentiation event took place approximately 10.5 Kyr BP for the two populations to diverge from their common ancestral lineage, aligning with the notion of a recent recolonization event of the Japanese population (Wang et al. 2016). Rising sea levels after the Younger Dryas Stadial (12.7–11.9 Kyr BP) led to an increase in the ocean distance between east (Ariake Sea) and west (China coastal waters) coasts of the East China Sea, serving as a vicariant barrier that prevented the dispersal of fishes between the two regions (Han et al. 2015). However, for the migratory species *I. elongata* , ocean distances between Ariake Sea and China coasts cannot simply account for the barrier to genetic flow among populations, as evidenced by the asymmetrical migration observed between the northern population and the Japanese population (Table S1). Previous studies (Wang et al. 2021a, 2021b; Zhang and Takita 2007) have identified Ariake Sound as a crucial spawning area for *I. elongata* along the east coast of the East China Sea. Hence, the split between the northern population and the Japanese population could be attributed to the different spawning ground preferences of the two populations alongside the substantial distance between the east and west coasts of the East China Sea.

Post‐Younger Dryas Stadial transgression not only shaped the contemporary coastline of the Northwestern Pacific (Wang and Wang 1982; Xie et al. 1996) but also facilitated secondary contact between the southern population and the northern population. According to our demographical estimation, migrations between those two populations began at 8 Kyr BP (Figure 4), coinciding with the key geological events of the submergence of the Dongshan land bridge and the reopening of the Taiwan Strait (Huang and Yu 2003).

### 4.3 Prospect on Fishery Management and Conservation of *I. elongata* Stocks

As previously shown, our genetic‐based groups identified here defined four *I. elongata* biological units along the northwestern Pacific coast. However, recent contraction in global effective population sizes portends an unpleasant status of *I. elongata* stock along the northwestern Pacific coast (Figure 4 and Table S1).

Our findings revealed two sympatric *I. elongata* populations in the waters of the South China Sea, Taiwan Strait, and East China Sea. These populations exhibit markedly different demographics and abundances. Over the past decade, those regions contributed over 60% of the annual landing of this species (CSY 2023). However, current harvest management frameworks prefer geographical‐based assessment rather than genetic stocks assessment. Such discrepancy between current management stocks and independent genetic units cannot result in a sustainable fishery. Previous practical studies on other commercial fishery targets, such as Atlantic cod ( *Gadus morhua* ), provide a good illustration of how the genetic marker‐based management program eases the dilemma of mixed‐stock fishery by enabling a “real time” fishery assessment framework (Dahle et al. 2018). Hence, with the aid of our or previous works, stock‐specific fishery reassessment as well as establishment of population‐specific quotas for *I. elongata* for each of the two populations should take action.

On the other hand, molecular tools also showed outstanding performance in assisting the establishment of conservation efforts. Both previous study (Wang et al. 2016) and our works uncover a genetic distinct lineage that only contains individuals from the Yalu River Estuary, indicating this lineage might be a cryptic species. However, considering the moderate genetic differences (*F*_ST_) to its congener from other regions (Table 3), recent divergence (Figure 4) and, most importantly, lack of morphological diagnosis that differs it from other genus *Ilisha* species, we hesitate to consider it as an independent species. Nevertheless, the Yalu River Estuary population, which demonstrates the lowest heterozygosity (0.1644) and smallest effective population size (*N*_e_ = 286) among all populations and harbors four private alleles, should be prioritized for conservation. As the relict of an ancient lineage among the *I. elongata* stocks, further research on this population could offer valuable insights into the adaptive potential of *I. elongata* stocks along the Northwestern Pacific Coast. Collaborative efforts should be formed between China and North Korea to protect this special stock of *I. elongata* , particularly at the river mouth of the Yalu, the breeding ground of the YE population. For instance, a special fishing ban could be instituted during the breeding season to protect *I. elongata* and other species. Furthermore, private alleles and other population‐defining SNPs identified by our works can be implicated as a powerful genetic tool for assessing the detailed distribution range of this unique population that enables the establishment of Multiple‐Use Marine Protected Areas (MMPAs) in the upcoming future.

## 5 Conclusion

Our analyses based on the enriched nuclear loci provided a comprehensive overview of the genetic diversity and population structure of *I. elongata stocks* along the Northwestern Pacific Coast. This commercially targeted fish species exhibits a degrowth in global stocks as proposed by the demography simulation result. Despite the minimal inter‐population differentiation among various geographical groups, four distinct populations of *I. elongata* were distinguished: the southern population, the northern population, the Japanese population, and the Yalu population. The current spatial distribution of these four populations may have been influenced by directional selection, geographical barriers, and the location of spawning grounds.

## Ethics Statement

All animal procedures performed in this research were done in accordance with the “Ethical Standards of the Shanghai Ocean University (2020)”.

## Conflicts of Interest

The authors declare no conflicts of interest.

## Supporting information


**Figure S1:** Six tested demographic models. Model 1, divergence event between pop 2 and pop 0 occurred prior to divergence between pop 1 and pop 0 under the scenario of strict isolation (SI); model 2, divergence event between pop 2 and pop 0 occurred posterior to divergence between pop 1 and pop 0 under SI scenario; model 3, pop 2 as a sub‐divergent split from pop 1 after the separation between pop 1 and pop 0 under SI scenario. Model 4–6 are designed on the basis of model 3 under three different divergence scenarios: Isolation‐with‐Migration (IM, model 4), Ancient Migration (AM, model 5), and Secondary Contact (SC, model 6). Arrows denote the asymmetric gene flow between geographically adjacent populations and red dashed lines denote the gene flow barriers. The highlighted model 6 was chosen as the best model.

**Figure S2:** PCA of the data matrix excluding all individuals from Yalu River Estuary (DD, CL1133 and CL1134). Each standalone dot denotes an individual. The color of the dot represents the geographic region, and the shape of the dot represents the sampling locality.

**Figure S3:** PCA of localities with more than 5 individuals. Each standalone dot denotes an individual. The color of the dot represents the geographic region, and the shape of the dot represents the sampling locality.

**Figure S4:** PCA of the data matrix with outlier exclusion Each standalone dot denotes an individual. The color of the dot represents the geographic region, and the shape of the dot represents the sampling locality.

**Table S1:** Simulation results of six demographic models based on site frequency spectrum (SFS) using FASTSIMCOAL2.8.

**Table S2:** Outliers uncovered by FDIST method implemented in ARLEQUIN across all populations after FDR correction (*p* < 0.05).

**Table S3:** Outliers uncovered by BAYESCAN across all populations (*q*‐value < 0.05).
Supporting Informations associated with this article can be found in the Dryad. DOI: https://doi.org/10.6084/m9.figshare.28342883.

## Data Availability

Raw sequence reads are available from the NCBI Sequence Read Archive (SRA), under BioProject PRJNA1122400.
