# Supplementary figures and images for: Population Patterns and Dynamics of Ilisha elongata (Clupeiformes: Pristigasteridae) Revealed by Target Enrichment Data
Source: Evol Appl. 2025 Aug 6;18(8):e70142. doi: 10.1111/eva.70142 (PMC12329003; doi:10.1111/eva.70142)

**Model 1**

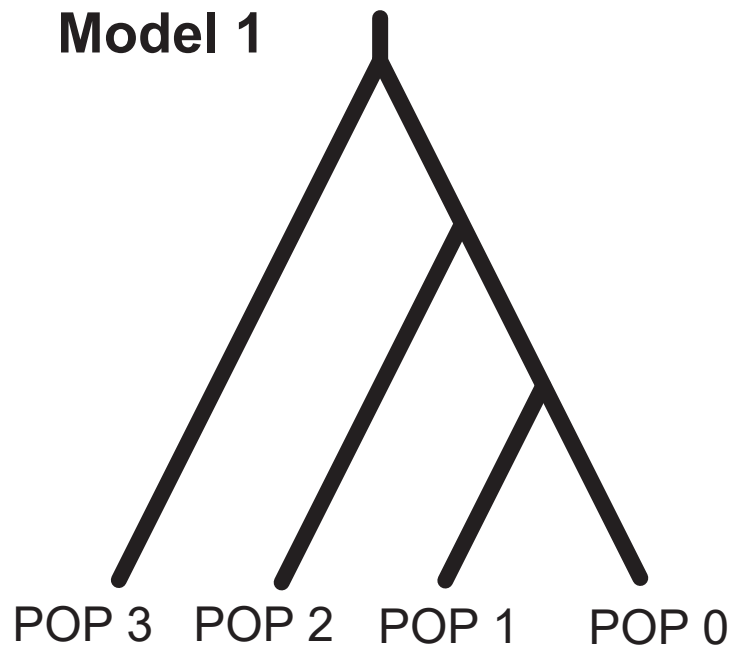

**Model 2**

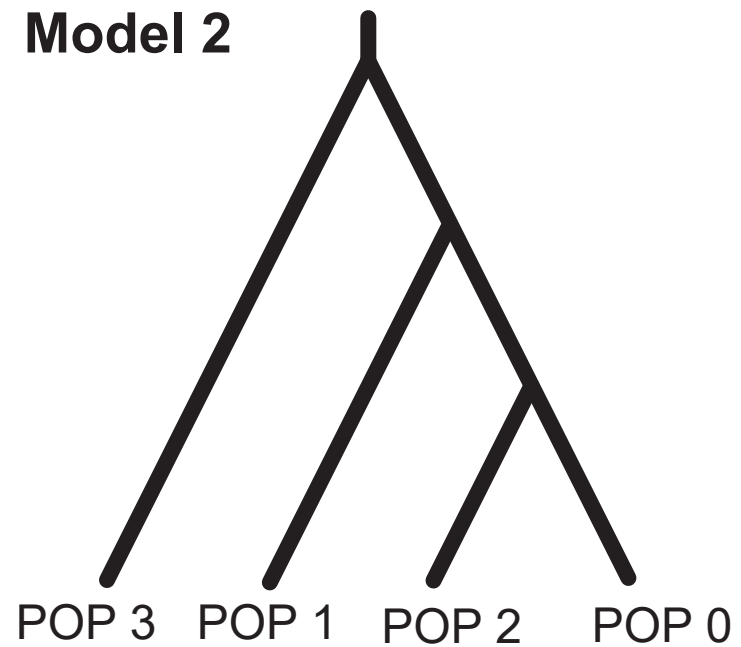

**Model 3**

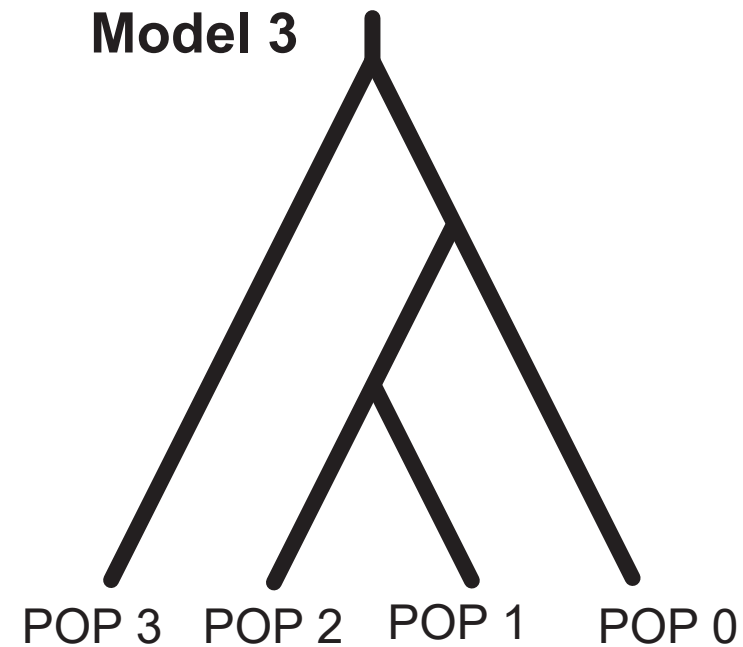

**Model 4  
(IM)**

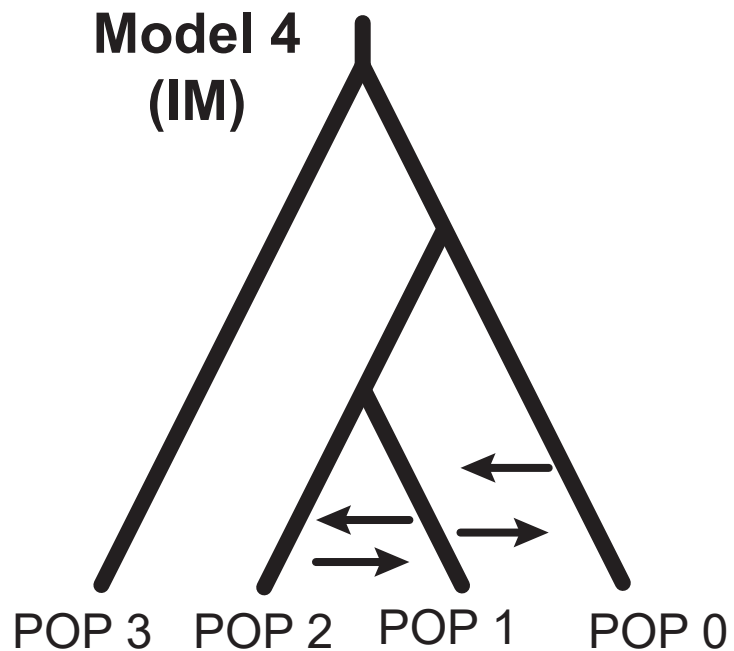

**Model 5  
(AM)**

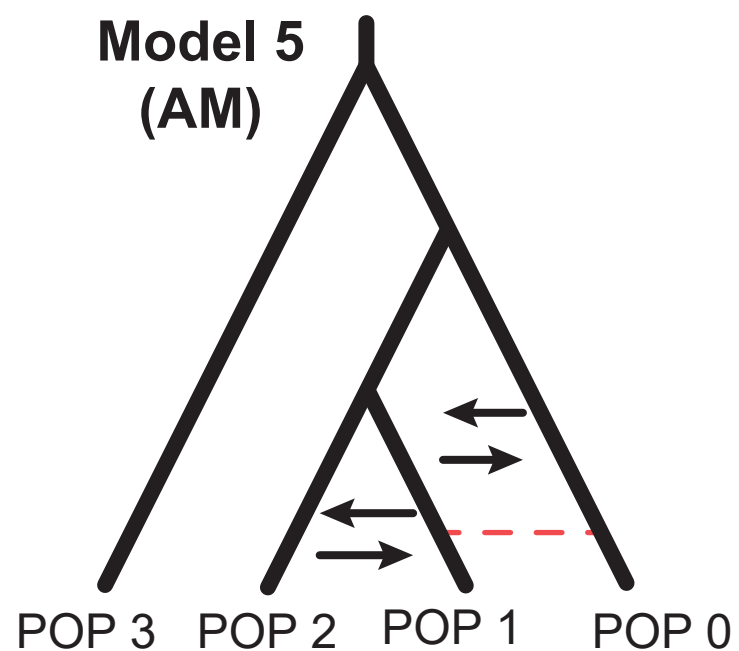

**Model 6  
(SC)**

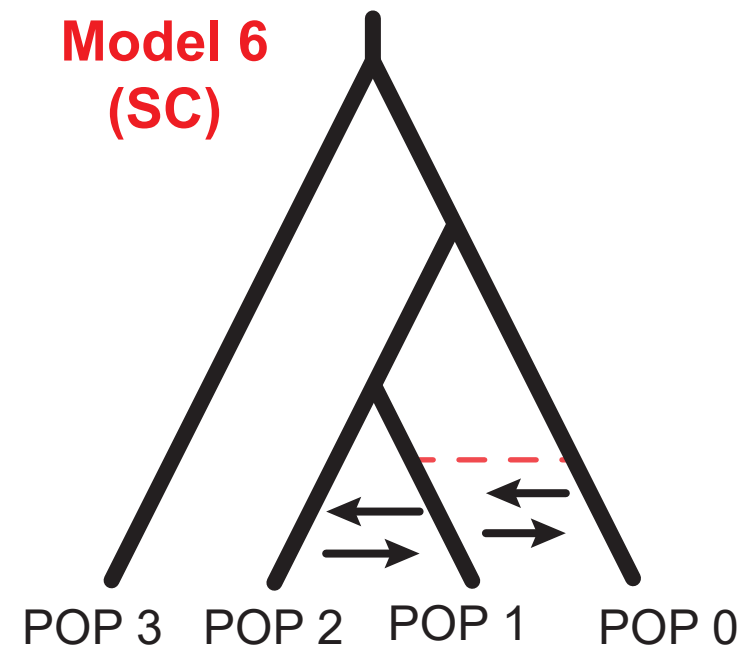

Supplement: Supplementary file 1 — Figure S1: Six tested demographic models. Model 1, divergence event between pop 2 and pop 0 occurred prior to divergence between pop 1 and pop 0 under the scenario of strict isolation (SI); model 2, divergence event between pop 2 and pop 0 occurred posterior to divergence between pop 1 and pop 0 under SI scenario; model 3, pop 2 as a sub‐divergent split from pop 1 after the separation between pop 1 and pop 0 under SI scenario. Model 4–6 are designed on the basis of model 3 under three different divergence scenarios: Isolation‐with‐Migration (IM, model 4), Ancient Migration (AM, model 5), and Secondary Contact (SC, model 6). Arrows denote the asymmetric gene flow between geographically adjacent populations and red dashed lines denote the gene flow barriers. The highlighted model 6 was chosen as the best model. [file EVA-18-e70142-s002.pdf]

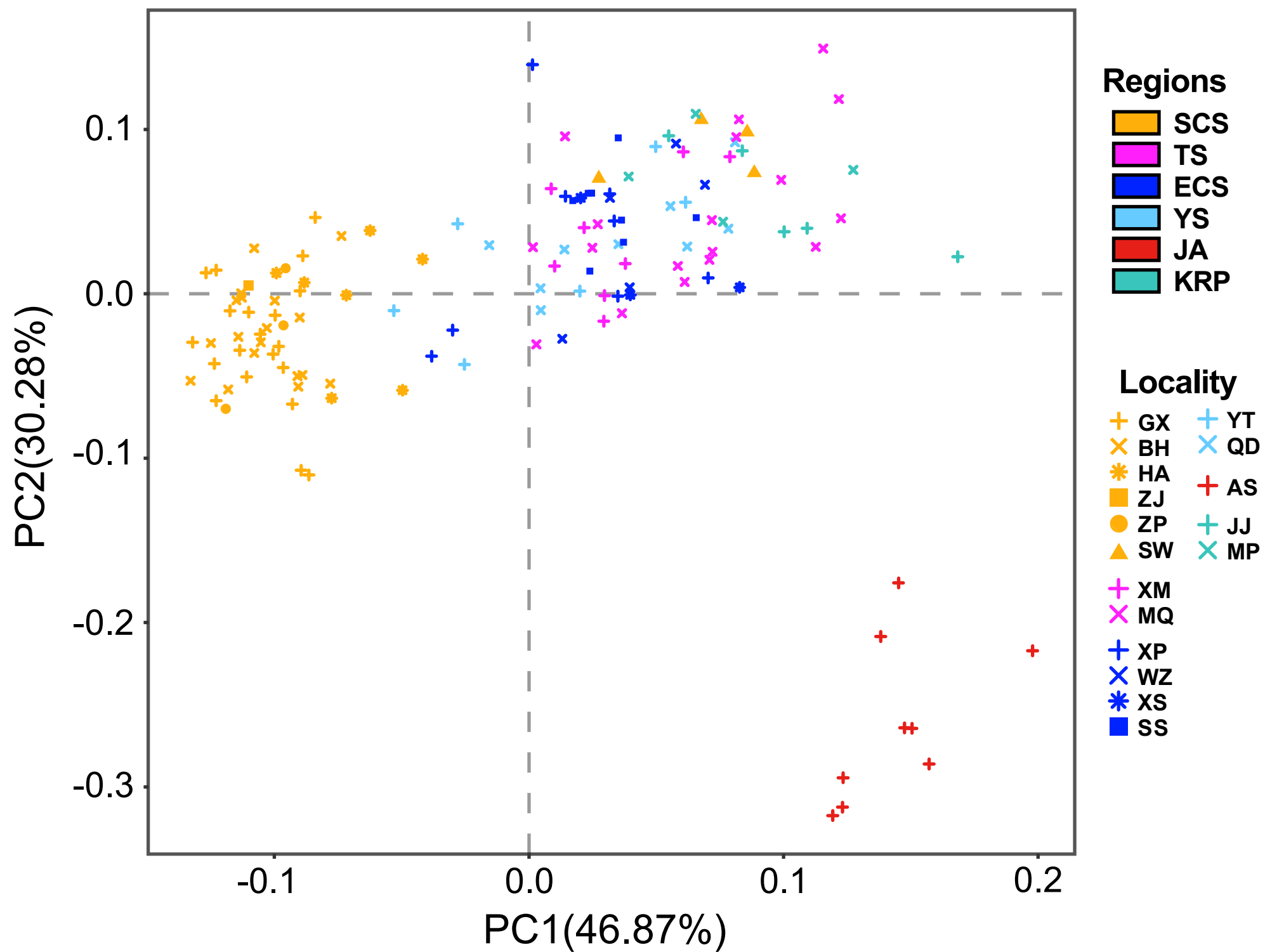

Supplement: Supplementary file 2 — Figure S2: PCA of the data matrix excluding all individuals from Yalu River Estuary (DD, CL1133 and CL1134). Each standalone dot denotes an individual. The color of the dot represents the geographic region, and the shape of the dot represents the sampling locality. [file EVA-18-e70142-s005.pdf]

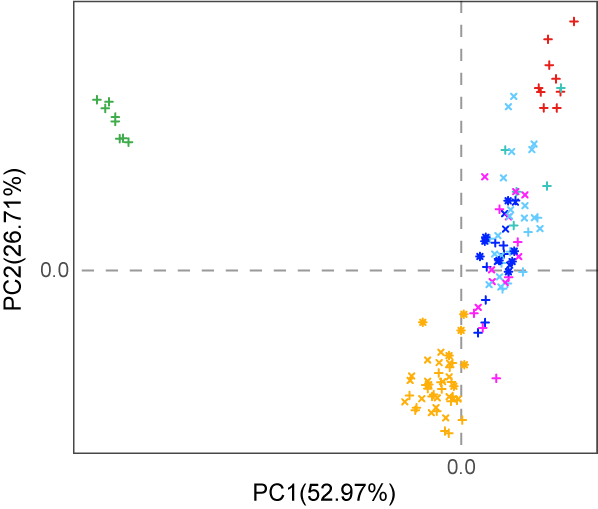

Supplement: Supplementary file 3 — Figure S3: PCA of localities with more than 5 individuals. Each standalone dot denotes an individual. The color of the dot represents the geographic region, and the shape of the dot represents the sampling locality. [file EVA-18-e70142-s007.png]

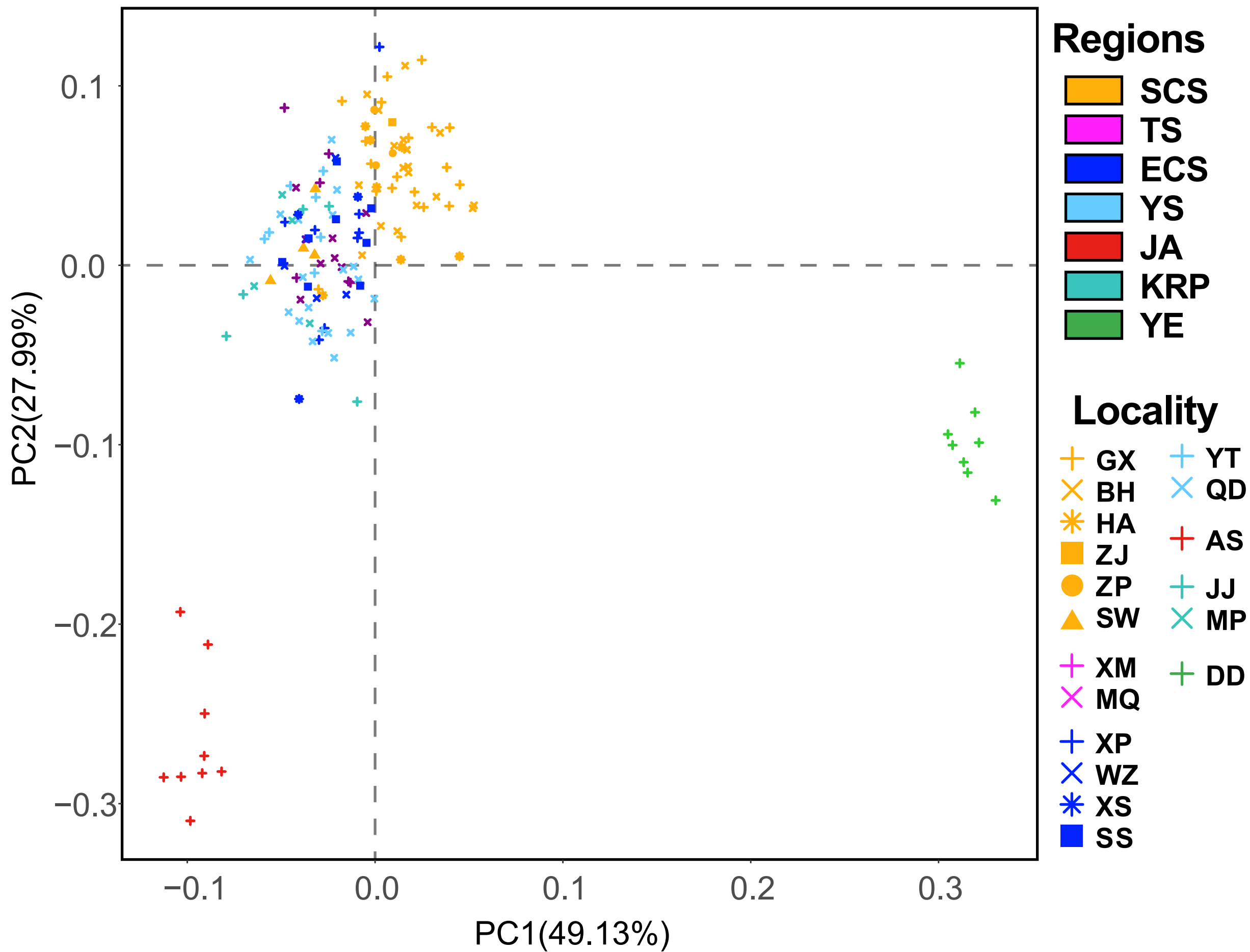

Supplement: Supplementary file 4 — Figure S4: PCA of the data matrix with outlier exclusion Each standalone dot denotes an individual. The color of the dot represents the geographic region, and the shape of the dot represents the sampling locality. [file EVA-18-e70142-s004.pdf]
